# Advances in Polypyrrole Nanofiber Composites: Design, Synthesis, and Performance in Tissue Engineering

**DOI:** 10.3390/ma18132965

**Published:** 2025-06-23

**Authors:** Lu Hao, Demei Yu, Xinyu Hou, Yixuan Zhao

**Affiliations:** 1 Department of Materials Engineering, Shaanxi Polytechnic Institute, Xianyang 712000, China; 18691006725@163.com (X.H.); 13098159434@163.com (Y.Z.); 2 Key Laboratory of Shaanxi Higher Education Institutions for Ultra-Flexible Forming Technology of Electronic Glass, Shaanxi Polytechnic Institute, Xianyang 712000, China; 3 State Key Laboratory of Electrical Insulation and Power Equipments, MOE Key Laboratory for Non-Equilibrium Synthesis and Modulation of Condensed Matter, School of Chemistry, Xi’an Jiaotong University, Xi’an 710049, China; dmyu@xjtu.edu.cn

**Keywords:** PPy, electrospun nanofibers, composite, tissue engineering

## Abstract

This review is different from previous studies focusing on polypyrrole (PPy) in universal fields such as sensors and supercapacitors. It is the first TO systematically review the specific applications of PPy-based electrospun nanofiber composites in the biomedical field, focusing on its biocompatibility regulation mechanism and tissue repair function. Although PPy exhibits exceptional electrical conductivity, redox activity, and biocompatibility, its clinical translation is hindered by processing challenges and poor degradability. These limitations can be significantly mitigated through composite strategies with degradable nanomaterials, enhancing both process compatibility and biofunctionality. Leveraging the morphological similarity between electrospun nanofibers and the natural extracellular matrix (ECM), this work comprehensively analyzes the topological characteristics of three composite fiber architectures—randomly distributed, aligned, and core–shell structures—and elucidates their application mechanisms in nerve regeneration, skin repair, bone mineralization, and myocardial tissue reconstruction (e.g., facilitating oriented cell migration and regulating differentiation through specific signaling pathway activation). The study further highlights critical challenges in the field, including PPy’s poor solubility, limited spinnability, insufficient mechanical strength, and scalability limitations. Future efforts should prioritize the development of multifunctional gradient composites, intelligent dynamic-responsive scaffolds, and standardized biosafety evaluation systems to accelerate the substantive translation of these materials into clinical applications.

## 1. Introduction

In the past few decades, one-dimensional nanostructures with nanoscale and molecular properties have made significant progress and are constantly meeting people’s needs, such as carbon nanotubes, inorganic semiconductors and metal nanotubes/wires, conjugated polymer nanofibers/tubes, etc. [1,2,3,4]. These nanostructures have a profound impact on the basic research and potential applications of nanoelectronics or molecular electronics, nanodevices, nanocomposites, biological nanotechnology, and medicine [1,2,3,4].

Conductive polymers (CPs) are a special type of conjugated polymers with inherent conductivity. Due to their unique polymer properties, excellent electrical and mechanical properties, stability, and biocompatibility, they have broad application prospects in fields such as energy storage [5], flexible electronics [6], and bioelectronics [7]. Polypyrrole (PPy) is a kind of biocompatible conductive polymer. Due to its excellent conductivity, excellent redox properties, biocompatibility, easy synthesis, and environmental stability, it has potential applications in the biomedical field [8,9]. In the field of drug delivery, PPy nanofiber composites enable precise controlled release through electrochemical stimulation or environmental responsiveness (e.g., pH/temperature changes). The high specific surface area of their nanofibrous structure significantly enhances drug loading efficiency while reducing systemic toxicity. In biosensing applications, these materials allow the construction of highly sensitive detection systems by immobilizing glucose oxidase or modifying specific aptamers, facilitating the development of portable diagnostic devices and laboratory-on-a-chip technologies. Their photothermal antibacterial properties, combined with sustained antimicrobial agent release, provide effective anti-biofilm solutions for surgical sutures and implant surfaces. Additionally, in medical imaging, these composites serve as multifunctional contrast agents, enhancing MRI contrast via superparamagnetic particle loading or enabling deep-tissue visualization through photoacoustic effects. In flexible wearable devices, self-powered electronic skins leveraging their high tensile strain sensitivity have been developed to monitor real-time electrocardiogram (ECG), electromyogram (EMG) signals, and sweat metabolites.

In recent years, the research on PPy in the field of tissue engineering has attracted particular attention. PPy can regulate cell activities, including electrical stimulation (ES) to synthesize deoxyribonucleic acid (DNA), migration, and proliferation of cells in the biological environment [10]. It was found that conductive PPy-based scaffolds could support cell adhesion and proliferation regardless of whether electrical stimulation was applied, but electrical stimulation significantly enhanced cell activity. In addition, PPy-based scaffolds with electrical stimulation significantly increased the expression and secretion of nerve growth factor and brain-derived neurotrophic factor [11]. However, PPy has an amorphous structure and is insoluble, making it difficult to process, and has poor degradability. Composites with degradable nanomaterials can solve this problem. Due to the similarity between nanofibers and natural extracellular matrix (ECM), the preparation of nanofiber scaffolds by combining PPy and other materials is the main research topic in tissue engineering.

Electrospun nanofibers are an important class of materials in the current research of tissue engineering scaffold materials [12,13], mainly due to their following characteristics: (1) Electrospun nanofiber films have a good porous structure, high surface volume ratio, and space volume. These properties facilitate the loading and release of bioactive molecules, such as proteins, nanomedicines, nucleic acids, etc. Moreover, a higher surface area is conducive to the contact and adhesion between cells and scaffold materials; (2) The electrospinning device can be used to produce fibers with diameters of tens of microns or even several nanometers. Nanoscale fibers are similar to the ordered multi-layer fiber structure of natural extracellular matrix. As a scaffold material, it can simulate ECM well, which is conducive to cell adhesion and proliferation on the surface. A single polymer or a variety of polymer composites can be fabricated into nanofiber materials by electrospinning technology. These polymers include a large number of natural polymers, synthetic polymers, and their composites to meet the needs of different tissue engineering materials.

The doping process of PPy regulates its conductivity, mechanical properties, and biological activity by introducing counter ions (such as Cl^−^, p-toluenesulfonate) or functional molecules (drugs, growth factors). The dopants are divided into inorganic materials (metal ions, carbon-based nanoparticles to enhance conductivity and mechanical strength), biomolecules (heparin, drugs to achieve anticoagulant or controlled release), and composite systems (such as Ag/dexamethasone synergistic antibacterial and anti-inflammatory), and are integrated into the scaffold through oxidative polymerization (oxidant selection, reaction medium optimization) and electrospinning process (in situ/post-treatment doping). Doping not only enhances interfacial adhesion (such as a high degree of deacetylation of chitosan to enhance PPy adhesion) but also achieves cell response regulation (such as neural differentiation) or stimulation-triggered drug release through electroactivity. However, it is necessary to balance the stability and biocompatibility of dopants and explore multi-mode response strategies to promote clinical application.

**Figure 1:**
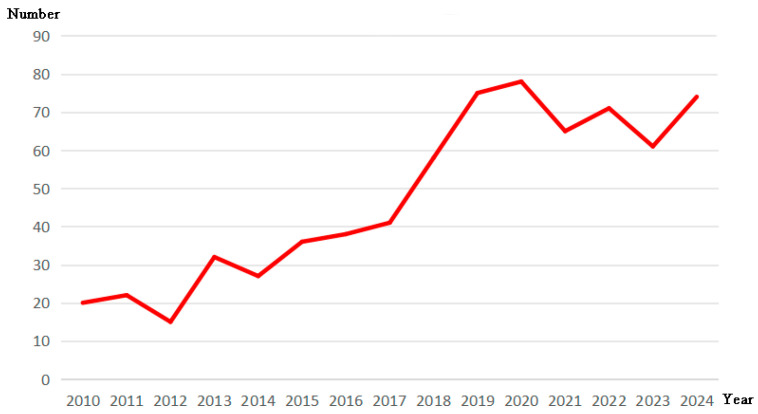
Number of papers published each year on the application of polypyrrole nanocomposites in tissue engineering.

This paper utilizes the Web of Science database and employs “PPy” and “tissue engineering” as keywords to screen over 100 relevant SCI papers with successful cases published between 2010 and 2024. The number of published papers per year from 2010 to 2024 (the abscissa is the year, and the ordinate is the number of papers) is also provided in Figure 1. It can be seen from the figure that since 2010, the number of papers related to research has generally increased year by year.

This paper mainly summarizes the research progress of PPy-based electrospun nanofibers as scaffold materials in the field of tissue engineering in recent years. PPy-based electrospun nanofiber composites mainly include three types of structures: randomly distributed PPy-based nanofibers, aligned PPy-based nanofibers, and core–shell structure PPy-based nanofibers. Then, the application of PPy-based electrospun nanofiber composites as scaffold materials in the fields of nerve, skin, bone, skeletal muscle, and myocardium is summarized.

## 2. Structural Classification of PPy-Based Electrospun Nanofiber Composites

Although PPy has great potential in tissue engineering applications, PPy still has some limitations related to its inherent properties. Although the main benefit of adding PPy to the scaffold is the injection of electroactive properties, for in vivo implants, PPy-based scaffolds must also have mechanical stability, biocompatibility, and biological activity. The concept of using pure PPy and ES in tissue engineering has been reported to facilitate cell maturation. However, they are limited in the form of thin films, and there are no data on their mechanical properties and biocompatibility; therefore, pure PPy is not suitable for in vivo implants [14]. In addition, it is known that CPs are very brittle; thus, it is difficult to use high-concentration CPs to fabricate conductive scaffolds [15]. A common strategy to overcome its inherent brittleness is to mix a small but sufficient amount of CPs with non-conductive polymers (such as PLA, PCL, chitosan, etc.) to simulate the conductivity of natural tissues. These polymers have less brittleness than CPs as a matrix, thus forming composite materials. In turn, the introduction of CPs helps to improve the moderate mechanical strength and Young‘s modulus of non-conductive polymers, which are usually too low to be used as biological scaffolds. In this sense, CPs can be regarded as filler particles (i.e., load-bearing media) in composites under the premise that there is sufficient interaction between CPs and polymer matrix interfaces, which will help to strengthen the mechanical properties of non-conductive polymer matrix (i.e., load-bearing media). The discussion in this review focuses on the preparation of PPy polymer composites using electrospinning technology, because it is the most common type of PPy-based electroactive scaffolds so far.

### 2.1. Randomly Distributed PPy-Based Nanofibers

**Figure 2:**
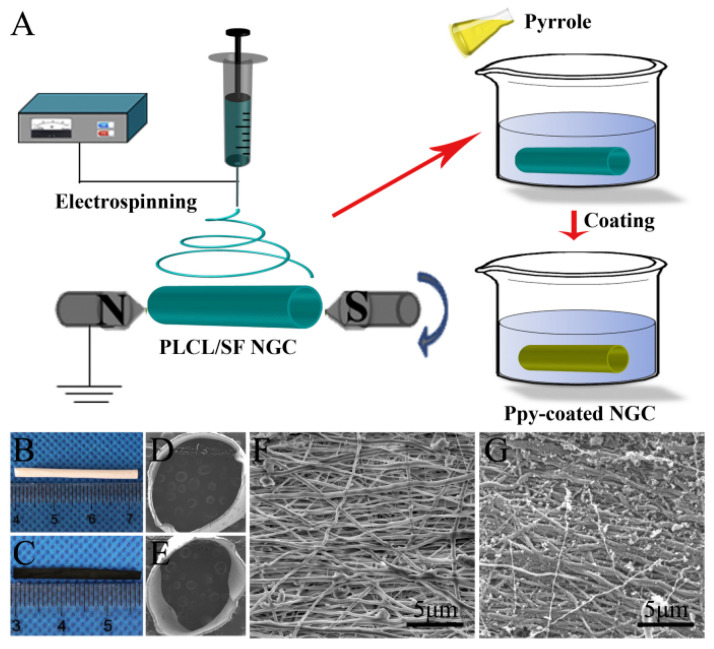
(**A**) Schematic illustration of Ppy-coated PLCL/SF NGC by electrospinning and in situ polymerization. Digital photos of (**B**) PLCL/SF NGC and (**C**) Ppy-coated NGC. Scanning electron microscope images of (**D**) PLCL/SF NGC cross-section, (**E**) Ppy-coated NGC cross-section, (**F**) the nanofiber surface of PLCL/SF NGC, and (**G**) the nanofiber surface of Ppy-coated NGC [21]. Copyright 2019 Materials Science and Engineering: C.

Randomly distributed PPy-based nanofibers are the most common type of PPy composite nanomaterials prepared by electrospinning [16,17,18,19,20,21]. Roca et al. [20] prepared PPy-coated polylactic acid (PLA) conductive nanofiber membranes and studied the effects of different reaction parameters on their physicochemical and dielectric properties. It is found that the conduction mechanism is based on the transformation law of PPy content: for the samples without PPy, there is a temperature-dependent relaxation process; for samples with high PPy content, a temperature-dependent conduction process; and for samples with low PPy content, a combination of these two processes. A uniform and continuous coating was obtained from 23 wt% PPy, and the percolation effect near 27 wt% PPy was observed. A higher percentage of PPy allows for higher conductivity (above 0.20 S cm^−1^), but aggregates appear in 34 wt% of PPy. Sun et al. [21] coated PPy on the surface of electrospun poly (l-lactic-ε-caprolactone)/silk fibroin (PLCL/SF) to prepare a PPy-coated nerve-guided catheter (NGC). The preparation process is shown in Figure 2.

### 2.2. Aligned PPy-Based Nanofibers

**Figure 3:**
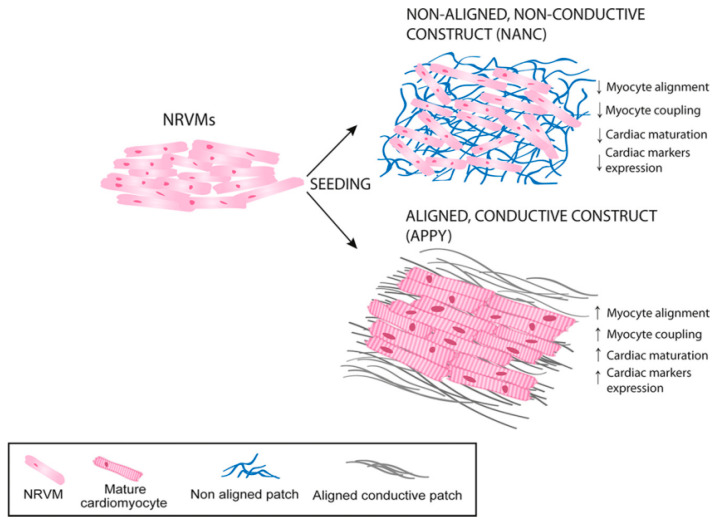
The growth of NRVMs on aligned and randomly distributed nanofiber scaffolds [22]. Copyright 2022 Nanomedicine: Nanotechnology, Biology and Medicine.

The application of aligned nanofibers in tissue engineering is mainly to control the growth direction of cells. The aligned nanofiber scaffolds can be used for tissue engineering research, such as artificial blood vessels and ligaments [22,23,24,25,26,27,28,29]. Mancino et al. [22] proposed an electrospinning structure of a new type of conductive composite fiber with oriented distribution combining gelatin, polylactic acid-glycolic acid, and PPy, which can be used as a heart patch. Three different types of cells (neonatal rat ventricular myocytes (NRVM), human lung fibroblasts (MRC-5) and induced pluripotent stem cells (iPSCs)) were used to study the cell-biomaterial interaction. According to its phenotype, all cell types show good viability and a unique structure on the surface of the construct. The growth of NRVMs on aligned and randomly distributed nanofiber scaffolds is shown in Figure 3.

**Figure 4:**
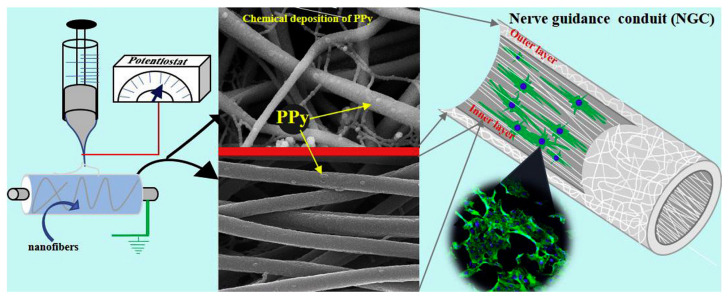
The equipment and SEM images for the preparation of aligned polypyrrole nanocomposite fibers [30]. Copyright 2018 Carbon.

Studies have shown that the proliferation and differentiation rate of cells on aligned PPy-based nanofiber scaffolds is faster than that of randomly distributed nanofiber scaffolds [30,31,32,33]. Shrestha et al. [30] designed a self-electrically stimulated double-layer nerve guidance catheter (NGC), which was realized by chitosan-grafted polyurethane and well-dispersed functionalized multi-walled carbon nanotubes (fMWCNTs) nanofiber mats after uniform coating of PPy. The NGC is assembled by an electrospinning mat, with a directional arrangement of the inner layer, covering a randomly oriented outer layer. The interconnected NGC structural framework exhibits a cell-biomaterial interface and improves physical and chemical properties, including electrical conductivity, mechanical strength, and cell compatibility. As a natural host matrix for natural extracellular matrix (ECM), it plays an important role in neural tissue engineering. During in vitro cell culture, the regeneration, proliferation, and migration of Schwann cells (S42) and the differentiation of rat pheochromocytoma cells (PC12) were greatly accelerated on the mats with aligned nanofibers compared with the mats with randomly oriented nanofibers. The morphology and phenotype of the spontaneous growth of axon bundles are preferentially guided along the axis of aligned nanofibers, which maintains strong adaptability in axon regeneration. The equipment and SEM images for the preparation of aligned polypyrrole nanocomposite fibers are shown in Figure 4.

In the field of tissue engineering, many studies focus on the surface morphology of conductive materials. However, there is little research on how electrical stimulation of this material affects nerve regeneration. Elashnikov et al. [34] studied the effect of electrical stimulation on randomly arranged and uniaxially arranged Polypyrrole-coated cellulose acetate butyrate (PPy/CAB) nanofibers. Research has shown that uniaxially arranged nanocomposite fibers have a strong impact on cell morphology and adhesion, with or without electrical stimulation. In addition, fluorescence microscopy showed that cells electrically stimulated on PPy/CAB exhibited longer axonal growth.

### 2.3. Core–Shell Structure PPy-Based Nanofibers

**Figure 5:**
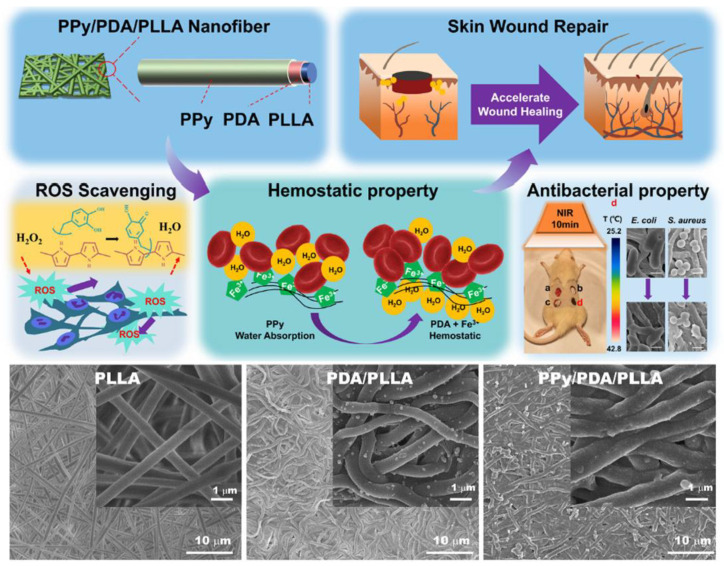
Mechanism of core–shell PPy/PDA/PLLA composites in the biological field [35]. Copyright 2022 International Journal of Biological Macromolecules.

Functionalized core–shell-structured composite nanofibers can be used to control the release of growth factors or drugs, as well as to develop high-sensitivity sensors and tissue engineering scaffold composites [35,36,37,38]. Xiong et al. [35] uniformly coated polydopamine (PDA) and PPy on the surface of poly (L-lactide) (PLLA) nanofibers by in situ polymerization to form a new PPy/PDA/PLLA three-layer core–shell structure. The schematics of the structural and mechanical properties of the new material are shown in Figure 5. The uniformly coated PPy and PDA layers can significantly improve the hydrophilicity, conductivity (Resistance was around 291 Ω), near-infrared photothermal antibacterial properties, wound hemostasis speed, antioxidant capacity, and active oxygen scavenging ability, respectively. In addition, PPy/PDA/PLLA nanofibers showed good biocompatibility. The rat wound healing model confirmed that PPy/PDA/PLLA nanofibers could significantly accelerate wound repair in vivo.

Coaxial electrospinning represents an advanced adaptation of conventional electrospinning technology, distinguished primarily by its implementation of a multi-lumen coaxial spinneret during the fabrication process. This innovative configuration allows two distinct material components to be simultaneously delivered through separate concentric channels, ultimately self-assembling into core–shell fibrous composites. The technique’s principal advantages lie in its capacity to achieve precise nanoscale control over sheath thickness, tunable mechanical integrity, and engineered degradation profiles—all while maintaining the inherent biocompatibility of the constituent materials. This unique combination of features enables customized fiber characteristics without compromising biological functionality, making it particularly valuable for biomedical applications requiring tailored material performance. Functionalized core–shell-structured composite nanofibers can be used to control the release of growth factors or drugs, as well as to develop high sensitivity. Zhang et al. [39] prepared lysine-doped PPy/regenerated spider silk protein (RSSP)/poly (L-lactic acid) (PLLA)/nerve growth factor (NGF) (L-PRPN) composite scaffolds by coaxial electrospray and electrospinning. This L-PRPN composite scaffold has a microfiber structure with a core–shell structure as the backbone and nanofibers as branches, and has good biocompatibility, cell adhesion, and relatively stable conductivity (the average conductivity was 16.70 ± 0.07 s cm^−1^).

In addition, to address the regulatory challenges arising from the complex interactions between processing parameters and material properties in electrospinning of polymeric tissue engineering scaffolds, Golbabaei et al. [40] proposed an innovative integration of SMILES molecular encoding and machine learning algorithms to construct a material-process-property prediction model. It achieves the first intelligent prediction of scaffold fiber diameter and conductivity and validates the model’s reliability through experimental verification. This strategy significantly narrows the optimization scope of process parameters and provides an efficient data-driven solution for optimizing electrospinning processes of diverse biopolymers.

## 3. Application of Electrospun PPy-Based Composites in Tissue Engineering

The electrical signals in the human body regulate various types of tissues, such as neural communication activity, heartbeat activity, bone regeneration, muscle contraction, and wound healing [41]. Due to the direct current and stable potential, the important role of bioelectric field leads to tissue repair and cell migration to the wound site [42]. PPy allows fine-tuning of chemical, electrical, and physical properties to meet the needs of the tissue parts using PPy [43]. In order to achieve a greater impact of electroactive scaffolds, their implementation should not only meet the general characteristics of biomaterials, but also meet the specific needs of each tissue that may change with other tissues due to differences in extracellular matrix and function. In addition to electrical conductivity, certain mechanical properties unique to each tissue should also be considered, such as the high compressive strength and modulus of bone tissue and the typical elasticity of skin tissue. Therefore, this section focuses on (i) the electrical properties of PPy-based electroactive scaffolds suitable for the specified tissue and its electrical stimulation phenomenon, and (ii) some other properties (such as mechanical properties) of PPy-based electroactive scaffolds related to the target tissue.

### 3.1. Bone Tissue Engineering

**Figure 6:**
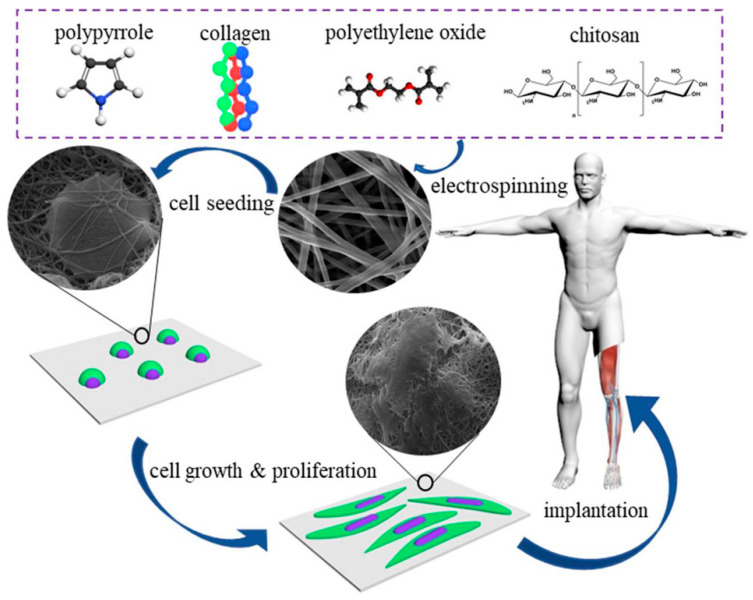
A conductive electrospun nanofiber scaffold containing conductive PPy polymer was fabricated to accelerate the healing of damaged tissues [45]. Copyright 2021 International Journal of Biological Macromolecules.

The electrical conductivities of cancellous bone and cortical bone were 1.6–2.0 × 10^−3^ S/cm and 5.8–6.3 × 10^−4^ S/cm, respectively. One of the common strategies to simulate the electrical properties of bone tissue is to add conductive fillers, such as PPy, to increase the conductivity of the scaffold [29,44,45,46,47,48,49,50,51]. Chitosan’s electroactive scaffold properties are tunable through molecular weight (MW) and degree of deacetylation (DD). A high DD (≥80%) boosts cationic charge density, enhancing electrostatic/coordination interactions with PPy or additives, while the controlled MW tailors porosity and degradation for optimized drug release. When combined with PPy via electrospinning, chitosan’s bioactivity and film-forming ability synergize with PPy’s conductivity. Zarei et al. [45] demonstrated this by fabricating PPy/chitosan/collagen scaffolds (0–25 wt% PPy). Conductivity increased with PPy content due to enhanced conductive particle contacts, yet all scaffolds exhibited low cytotoxicity from bioactive chitosan/collagen. Notably, the 10 wt% PPy scaffold achieved optimal performance, balancing electrical functionality and biocompatibility for cell proliferation. Liu et al. [49] self-polymerized dopamine (DA) on the surface of polylactic acid (PLLA)/hydroxyapatite (HA) nanowire composite fibers using electrochemical deposition to form an adhesive polydopamine (PDA) film, and constructed a stable silver nanoparticle (Ag-NPs) coating on it by electrochemically driven Ag^+^ coordination and PPy-mediated chelation to achieve a stable and slow release of Ag-NPs. The synthesis mechanism of composite fibers is shown in Figure 6. The PLLA/HA/PDA/PPy/Ag composite fiber promoted the nucleation and growth of apatite on the surface, and had good cell compatibility with osteoblasts, indicating the ability to induce osteogenic differentiation. In addition, the combination with silver ions serves to stabilize and control the slow release of Ag^+^, which contributes to the long-term antibacterial performance of the material.

In addition to increasing the concentration of PPy, the use of different PPy morphologies is also an attractive strategy to improve the electrical properties of electroactive scaffolds [52,53].

PPy is usually very fragile, with a Young’s modulus of 180 MPa, which is very low compared to the Young’s modulus of bone (especially cortical bone). This characteristic is contrary to the requirements of bone scaffolds, which require strength and ductility to avoid brittle fracture. In order to overcome this problem, the mechanical properties of PPy can be optimized by doping or combining PPy with metals, ceramics, or other polymers with higher mechanical properties than PPy. Maharjan et al. [54] prepared PCL/PPy composite nanofibers by electrospinning for bone tissue engineering scaffolds. The mechanical strength test of the PCL/PPy scaffold showed that the Young’s modulus (YM) of the composite was two to four times higher than that of the single PPy material, and the tensile strength (TS) was increased by three to four times. With the increase in PPy concentration in the composite scaffold, YM and TS also gradually increased.

Tissue scaffolds that allow the control of the behavior of their cells are of particular interest in tissue engineering. Hardy et al. [55] described the preparation of conductive fiber-based bone tissue scaffolds (electrospun polycaprolactone nonwoven mats with interpenetrating networks of PPy and polystyrene sulfonate) that can electrically stimulate human bone marrow mesenchymal stem cells and enhance ALP activity and calcium deposition. The application of electrospun PPy nanofibers in bone engineering is shown in Table 1.

### 3.2. Neural Tissue Engineering

**Figure 7:**
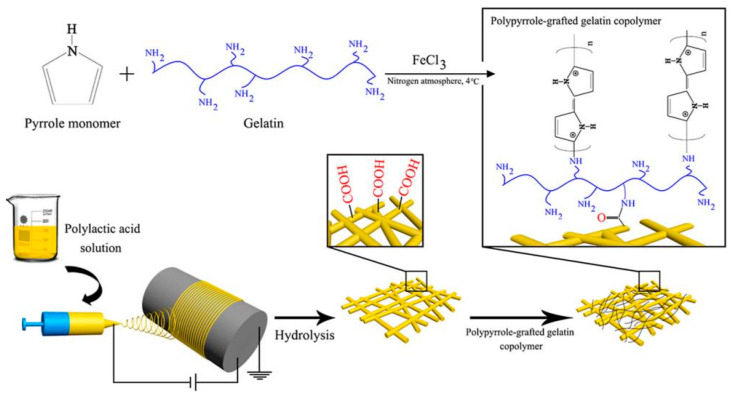
Schematic diagram for the preparation of gelatin/PPy/PLA nanofiber composite scaffolds [68]. Copyright 2021 Polymer.

Neural networks in the human body play a unique and important role in all physiological processes, including cell recognition, sensory, and motor functions. The application of electroactive scaffolds is attractive and promising for further promoting the growth and differentiation of neurons and the formation of neural networks [21,30,33,34,38,56,57,58,59,60,61,62,63,64,65,66,67]. The constructed PPy/SF conductive NGC, together with ES, can effectively promote axonal regeneration and myelin formation in vivo. In addition, it was found that the MAPKs signal transduction pathway was activated by ES at the conduction conduit. The mechanical properties of scaffolds for nerve tissue engineering should imitate the mechanical properties of ECM to promote the neural differentiation of cells. The use of conductive polymers in electroactive scaffolds can reduce or improve the mechanical properties of the scaffolds. Therefore, it is necessary to determine the optimal composition of the conductive polymer in the electroactive scaffold to obtain improved mechanical properties [68,69,70,71]. Imani et al. [68] chemically synthesized a gelatin-PPy conductive copolymer by grafting pyrrole with different weight ratios on the gelatin chain, and then grafted it onto electrospun PLA nanofibers to improve its conductivity and biological interaction. The preparation process of gelatin/PPy/PLA nanofiber composite scaffolds is shown in Figure 7. The research revealed that increasing the PPy content enhances the composite’s electrical conductivity, with the highest value (11.95 ± 1.10 mS/m) achieved at 20 wt% PPy content. Pheochromocytoma (PC12) cells were used to study the composite scaffold in vitro, including MTT and cell adhesion studies. The results show that the scaffolds containing 15% and 20% PPy can provide acceptable conditions for the adhesion and growth of nerve cells and can be introduced as a promising scaffold in nerve tissue engineering applications. Talebi et al. [70] prepared two kinds of graphene-containing PCL/gelatin/PPy and PCL/polyglycerol sebacate/PPy composite scaffold materials with intrinsic conductive properties using the electrospinning process and studied the effect of graphene on the properties of PCL/gelatin/PPy and PCL/polyglycerol sebacate/PPy. The results showed that the addition of graphene (3 wt%) significantly adjusted the physical and mechanical properties of the scaffold, increasing the conductivity from 0.1 to 3.9 ± 0.3 S/m, and the toughness was 76 MPa (PCL/gelatin/PPy) and 143.4 MPa (PCL/PGS/PPy). In addition, in the PCL/gelatin/PPy system, the elastic modulus of the scaffolds with 0,1 and 2 wt% graphene were reported to be 210, 300, and 340 kPa, and 72, 85, and 92 kPa in the PCL/PGS/PPy system.

Increased hydrophilicity contributes to the attachment of cells to the scaffold, thereby enabling better cell proliferation and differentiation [72,73,74]. Sadeghi et al. [72] developed a ternary PCL/chitosan/PPy composite scaffold using electrospun nanofibers, combining chitosan’s hydrophilicity and PPy’s electroactivity. The scaffold exhibited a 66% reduction in water contact angle due to chitosan, while PC12 cell studies revealed a 356% increase in proliferation and neurite extension compared to pure PCL. The synergy between high-DD chitosan-enhancing PPy interfacial adhesion to mitigate CP brittleness and PPy’s electroactivity (enabling real-time monitoring or drug release) underpinned the scaffold’s multifunctionality. By optimizing chitosan’s DD, MW, and PPy concentration, such composites balance electroactivity, mechanical resilience, and biocompatibility, advancing implants for tissue regeneration and therapeutic applications like electrically controlled drug delivery.

In order to further improve the processability, solubility, and hydrophilicity of PPy and enhance its further application in tissue engineering, our research group [75,76] prepared a series of PPy derivatives as composite scaffolds for electroactive materials, and applied them in the field of tissue engineering to study the effects of the surface morphology and electrical properties of the scaffolds on neural cell behavior.

**Figure 8:**
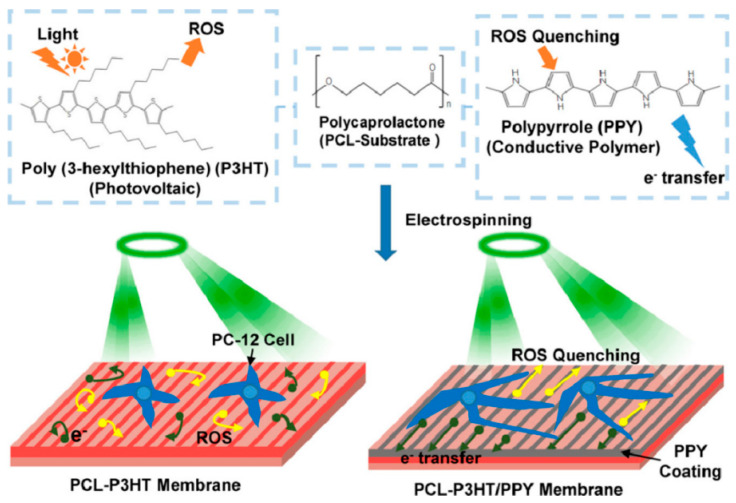
Preparation and photoelectric mechanism of P3HT/PCL/PPy heterojunction [77]. Copyright 2022 Acta Biomaterialia.

In addition, some researchers have used electrospun PPy-based composites for photoelectric biomaterials, which is a potential treatment option for neurodegenerative diseases (such as macular degeneration). Yuan et al. [77] developed a simple antioxidant, biocompatibility, and fibrous membrane heterojunction, which is composed of photosensitive polymer poly (3-hexylthiophene) (P3HT), PCL, and PPy. Compared with the traditional PCL-P3HT, the material not only has a lower impedance but also enhances the neurogenesis of PC-12 cells under light stimulation. Its mechanism is shown in Figure 8. This work demonstrates the importance of maintaining the PV-CP heterojunction while providing an optoelectronic platform for neural and optical tissue engineering. The application of electrospun PPy nanofibers in tissue engineering and regenerative medicine is shown in Table 2.

### 3.3. Skin Tissue Engineering

**Figure 9:**
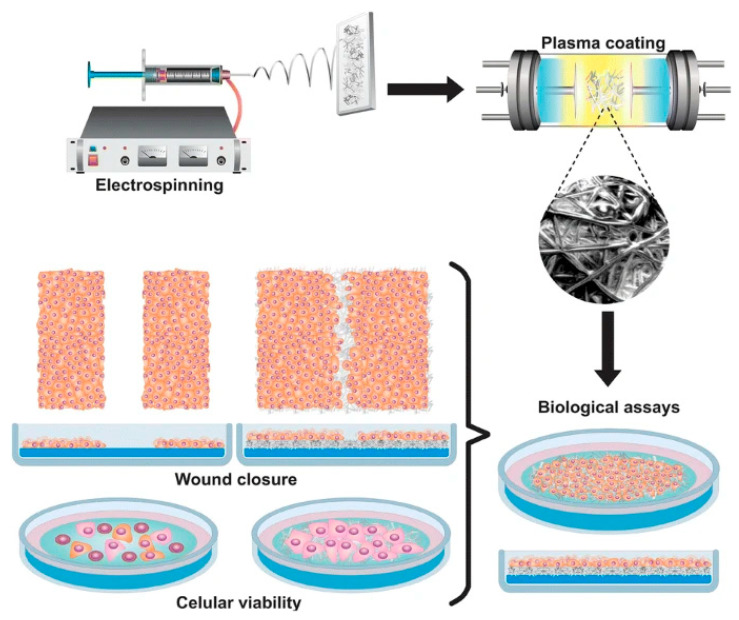
Electrospun the polypyrrole/iodine scaffolds of biopolymers used in skin tissue engineering to develop and create artificial skin tissue for replacement dermal substitutes [80]. Copyright 2019 Journal of Materials Science.

Skin trauma is one of the most common injuries caused by burns, diabetes, trauma, surgery, wound beds, and aging problems. Scaffold applications can expand ECM, provide potential opportunities for cell adhesion, proliferation, and migration, and, ultimately, enable new skin tissue regeneration (such as keratinocytes and fibroblasts). In the management of skin trauma, the development of electroactive scaffolds has a beneficial effect on enhancing the repair process of congenital trauma (such as local inflammation, cell infiltration, and neovascularization) [78]. In order to improve the electrical properties of skin scaffolds to meet the requirements of skin conductivity, researchers have conducted some research [79,80]. Massoumi et al. [79] prepared uniform, conductive, and biocompatible nanofiber scaffolds using an electrospinning PCL solution and synthesized PEGs-b-(PPy)_4_ copolymer (the conductivity was 0.24–0.31 S/cm). The properties of the prepared nanofibers as tissue engineering scaffolds were tested in terms of biological (biocompatibility and biodegradability) and physicochemical (electroactivity, electrical conductivity, mechanical properties, and morphology) characteristics. As a result, it was found that the prepared electrospun nanofibers were suitable scaffolds for tissue engineering applications requiring electroactivity. Roman-Doval et al. [80] prepared PPy/iodine-coated biopolymer scaffolds for skin tissue engineering using electrospinning technology and developed and manufactured artificial skin tissues for dermal substitutes (Figure 9). Studies have shown that the polypyrrole/iodine complex is a scaffold with the best coating for the growth of HaCaT cells, improving cell viability, adhesion, and cell healing.

In addition, Ma et al. [81] fabricated a conductive polypyrrole@keratin nanofiber membrane (PPy@KNM) with excellent biocompatibility and non-cytotoxicity via a combination of electrospinning and in situ chemical polymerization. This conductive membrane effectively accelerated cell growth and proliferation under electrical stimulation, demonstrating its suitability as a functional biomedical material for promoting wound healing.

### 3.4. Skeletal Muscle Tissue Engineering

Skeletal muscle tissue accounts for about 45% of the total weight of the human body and is responsible for generating the power of various biological motor functions. Muscles can be regarded as electromechanical actuators that convert electrical energy transmitted from the nervous system into mechanical energy. Skeletal muscle has the ability to regenerate from mild injury. However, severe injuries caused by major trauma or medical reasons (such as myopathy or long-term denervation) usually lead to irreversible loss of muscle function. Because of its ability to simulate the function of muscle tissue as an electromechanical actuator, PPy has gained attention in the development of electroactive muscle scaffolds. These actuators even exceed the performance of natural muscle tissue in terms of working density, which makes them replicate many muscle-like actions in vitro and in vivo [82,83]. Harjo et al. [82] made glucose–gelatin nanofiber scaffolds have electrical conductivity and electrical activity via the chemical and electrochemical deposition of PPy (doped with trifluoromethanesulfonic acid). Since it may be a potential electromechanically activated cell growth matrix, the linear driving properties of this material in cell culture medium (CCM) were studied. Based on the flux of the main Na^+^ ions from CCM, the material shows a relatively stable cation drive in CCM regardless of the deposition conditions.

The progress in tissue engineering and biomaterial development may provide scalable solutions to the problem of large skeletal muscle defects. Previous research on the development of skeletal muscle regeneration scaffolds has focused on strategies to increase electrical conductivity, which improves satellite cell attachment and differentiation. However, these strategies usually increase scaffold stiffness, and some studies have shown that this may be harmful to the development of myoblasts. Browe et al. [84] synthesized a PPy-PCL copolymer, which was designed to increase the conductivity of the scaffold without significantly affecting the stiffness. Different scaffolds were prepared using electrospinning, and their applicability to the proliferation and differentiation of myoblasts was evaluated. These scaffolds include aligned and randomly aligned pure PCL, 10% PPy-PCL, 20% PPy-PCL, and 40% PPy-PCL iterations. Only the scaffold containing 40% PPy-PCL has measurable electrical conductivity, and the addition of PPy-PCL has no significant effect on the stiffness of the scaffold. Compared with a pure PCL scaffold, the PPy-PCL copolymer significantly increased the adhesion of C2C12 myoblasts; however, the change in PPy-PCL concentration did not significantly change cell adhesion. In addition, by measuring the fusion index and the number of nuclei in each myotube, the scaffold with PPy-PCL promoted myoblast differentiation more than the scaffold made of PCL. In almost all measurements, the scaffolds composed of aligned nanofibers were superior to the scaffolds composed of randomly distributed nanofibers. These results suggest that electrical conductivity may not be a key factor in improving skeletal muscle scaffolds. On the contrary, the guidance cues of cell attachment and orientation may have a greater impact on myoblast differentiation.

### 3.5. Myocardial Tissue Engineering

The myocardium is a type of conductive tissue; therefore, conductive materials are used to simulate its inherent properties to repair damaged tissue. However, the inhibition of electrical conductivity may occur during the formation of fibrotic tissue or the remodeling of cardiac myocytes that damage cardiac function. Electroactive scaffolds are used as a strategy to help repair and improve the conductivity of the network to promote electrical connections between cells in the scaffold [85,86,87,88,89,90] Gelmi et al. [86] introduced an electroactive scaffold that provides electrical, mechanical, and topographic guidance for induced human pluripotent stem cells (iPS). The polylactic acid-glycolic acid fiber scaffolds coated with conductive PPy can provide direct electrical stimulation and mechanical stimulation to iPS. The electroactive scaffold has no cytotoxic effect on iPS, and the expression of cardiac biomarkers increases for both stimulation and non-stimulation regimens. This study is the first application of PPy as a supporting electroactive material for iPS and the first development of a fiber scaffold capable of dynamic mechanical drive.

The patch design for the treatment of heart disease should match the mechanical behavior of the myocardium. Olvera et al. [91] fabricated auxetic patches by melt electrospinning writing (MEW), which can overcome the limited elastic range of the traditional square patch design. Nutritional plaques can adapt to the strain and stress of the human myocardium during relaxation and contraction. The results show that the geometry of the auxetic patch can be fine-tuned to reflect the anisotropic mechanical properties. The effective stiffness anisotropy ratio (E-1/E-2) of the acrylic patch is consistent with the directionally dependent mechanics of the heart. In addition, the in situ polymerization of doped PPy on the auxetic patch imparts electrical conductivity (2.73 ± 0.3 S/m) similar to that of human myocardium.

**Figure 10:**
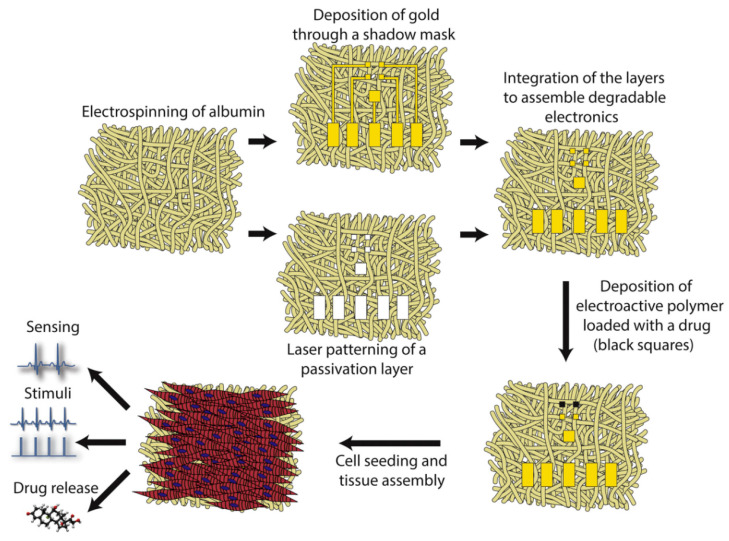
Schematic diagram of preparation of cardiac tissue engineering scaffold [92]. Copyright 2018 Journal of Controlled Release.

The ability to sense tissue function online, provide stimuli to control contractility, and effectively release drugs within the engineered tissue microenvironment can enhance tissue assembly and improve the therapeutic outcomes for implanted engineered tissues. In order to endow the cardiac patch with this ability, Feiner et al. [92] developed an elastic and biodegradable electronic scaffold. The preparation process and mechanism are shown in Figure 10. The scaffold is composed of electrospun albumin fibers, which are used as both the substrate of the evaporated gold electrode and the passivation layer. The myocardial cell tissue inoculated on the electronic scaffold becomes a functional heart tissue, and its function is recorded online. In addition, electronic scaffolds can activate engineered tissues to control their function and trigger drug release. After implantation, these electronic scaffolds degrade, leading to the dissociation of inorganic materials from the scaffold. The establishment of this technology can be used for various degradable devices for tissue engineering, as well as original cell-free devices for electronic components used in the short term in vivo. The integration with gold was achieved through direct deposition of gold electrodes onto electrospun fiber scaffolds. This configuration enables the recording of extracellular signals from engineered tissues while simultaneously controlling the contraction and release of the anti-inflammatory drug dexamethasone.

## 4. Conclusions and Outlook

This paper briefly reviews the main types of electrospun PPy nanocomposite fiber materials and their related applications in the field of tissue engineering. Discussions were conducted on preparation, physical, chemical, mechanical, and biological properties, as well as biomedical applications.

Electrospinning is a widely used technology and an ideal platform for preparing continuous ultrafine fibers. It has many advantages, and its performance can be adjusted by modification, which may solve the problem of the applicability of PPy and bring new application fields. In the past decade, the research on the preparation of PPy ultrafine fibers using electrospinning has developed rapidly. Although the direct electrospinning of pure PPy is difficult due to its low solubility and inherent brittleness, several strategies have been developed for the manufacture of PPy-based fibers, including the co-electrospinning of PPy with other spinnable carrier polymers and the synthesis of electrospun fiber templates, which are developed to solve this problem and improve the electrical, mechanical, thermal, and biological properties of electrospun PPy fibers. However, in order to further improve the performance of devices based on electrospun PPy materials and achieve the aforementioned interesting applications, many major challenges still need to be solved.

Firstly, due to the inherent properties of traditional CPs with low solubility and rigid frameworks, it is still a challenge to improve their solubility or spinnability by modifying or synthesizing new PPy derivatives, or finding/synthesizing better organic solvents or functional dopants. For example, the functional dopant sodium bis (2-ethylhexyl) sulfosuccinate (NaDEHS) can improve the solubility of PPy and form soluble PPy in organic solvents for electrospinning [93]. Ionic liquids are very stable and can be used as dopants or solvents. The improvement of solubility and processability of PPy in common organic solvents (including water and ionic liquids) is critical for more practical applications.

Secondly, in order to further improve the physical/chemical properties (e.g., thermal stability) and equipment performance (e.g., signal flow and stability) of electrospun PPy fibers, one approach is to fabricate polymers or nanofillers containing multiple functions, such as carbon nanotubes, graphene nanoplates, and metal or semiconductor nanoparticles/quantum dots, because polymer-based composites have shown improved performance and better applicability. In addition to adjusting the material composition or introducing functional materials during electrospinning, another important method is surface or chemical modification of PPy-based fibers through the adsorption of bioactive molecules, interception of small/large molecules, or covalent chemical coupling. This is an effective method to change conductivity, hydrophilicity/hydrophobicity, and biological activity, which is critical for neuroprobe and biosensor applications (e.g., surface modification of Au electrodes coated with PPy composite nanofibers by adsorbing ssDNA [94]).

Thirdly, electrospinning provides a multifunctional platform for the controllability of the morphology and structure of PPy fibers, which may bring new application fields. For example, core–shell nanofibers and hollow nanotubes synthesized using coaxial electrospinning or electrospun fiber templates can be used for drug delivery devices; spiral/wave or patterned fibers can be used for stretchable and flexible devices; the fiber array arranged using near-field electrospinning or nanofiber bundles can improve the performance of FET or bioactuator; 3D conductive fiber scaffold with controllable morphology, porosity, and pore size is suitable for tissue engineering applications. In addition, the reported electrospun PPy fibers/tubes usually exhibit a relatively large diameter from 100 nm to a few microns, and electrospun PPy nanofibers/tubes with an outer diameter of less than 100 nm are rarely reported. Therefore, precise control of the diameter, morphology, structure, and assembly of electrospun CP fibers remains a challenge in many cases of PPy.

Fourthly, the large-scale production of PPy-based fibers using electrospinning remains a challenge for commercial applications. At present, out of academic interest, most research on the manufacture and application of electrospun PPy fibers uses single-needle electrospinning in the laboratory. More attention should be paid to the engineering understanding and technical optimization of PPy fiber electrospinning process using various multi-needle/multi-nozzle electrospinning and nozzleless electrospinning technologies [95,96,97,98,99] to increase its application range. In addition, the poor mechanical properties and biocompatibility of PPy are additional challenges for regenerative medicine, especially for devices implanted in the human body. PPy composites containing other more biocompatible components are likely to exhibit better performance in biomedical applications. It should be noted that the biocompatibility of PPy composites depends on the type of additives and the synthesis method. For example, compared with metal nanoparticles prepared by traditional chemical methods, metal nanoparticles synthesized using green methods have much less cytotoxicity.

Finally, although there is compelling evidence that electrospun biopolymer fiber scaffolds have a significant impact, the manufacture of CPs has begun as biosensors, biological actuators, tissue engineering scaffolds, drug delivery devices, and neural interfaces. In order to support these important biological applications, two key issues must be addressed. The first problem is that the organic solvent is often used in solution electrospinning. The use of volatile and toxic solvents shows that cells or tissues cannot directly contact the electrospun structure without completely removing the solvent, which raises concerns about the cytotoxicity caused by residual solvents and solvent accumulation. The second problem is that the traditional method of electrospinning has challenges in the precise production and replication of structured 3D fiber scaffolds [100], which may affect the application of electrospun nanofiber scaffolds in engineering soft tissues. Melt-electrospinning (or melt-electrospinning writing) [101] is considered a promising candidate to address these two challenges, because melt-electrospinning nanofibers can be directly attached to cells without affecting their survival rate [102], and 3D (or even tubular) scaffolds with regular structures and high porosity (>85%) can be prepared by melt-electrospinning writing [101,103,104,105]. However, the challenge is that CPs are difficult to melt into electrospun fibers. Therefore, a combination of electrospinning and other manufacturing methods or post-processing (e.g., melt-electrospinning fiber template synthesis) may be a viable option. Due to the combination of the electroactivity of CPs, the biocompatibility of biopolymers, and good mechanical properties, electrospun CPs/biopolymer nanofibers are ideal candidates for tissue templates, artificial organ implants, and drug delivery that require electrical stimulation.

Tissue engineering relies heavily on the ability to expose cells that migrate to damaged tissue to precisely defined biophysical, biochemical, and biomechanical cues. By incorporating conductive PPy into the scaffold, a more cell-friendly tissue engineering construct can be developed. Although several studies have demonstrated the superiority of PPy in animal models, it has not yet been studied in humans. The main problem to be solved in the future is the huge gap between in vitro and in vivo outcomes. The research progress in this field may help researchers in this field to better predict the biological fate of PPy and reduce the gap between in vitro and in vivo results. A better transformation relationship between researchers, biotech companies, and clinicians is needed to elevate PPy-based conductive materials to a higher level and achieve clinical trials of these materials. Although it must be acknowledged that clinical trials are very expensive, researchers may consider using humanized animal models as an alternative test platform. The application of PPy-based nanofibers in surgical implants and tissue engineering remains challenging, including a mechanical-electroactive trade-off due to PPy’s inherent brittleness and low conductivity (<1 S/cm) from insufficient doping, coupled with poorly understood interfacial dynamic behaviors (e.g., fatigue under cyclic loading). Additionally, biocompatibility risks from dopant residues (e.g., oxidative byproducts, acidic ions) and the lack of long-term metabolic clarity hinder clinical translation, while existing systems’ reliance on single-mode electrical stimulation limits spatiotemporal regulation (e.g., growth factor gradients) or multimodal synergy (e.g., photo-electro-magnetic coupling). To overcome these limitations, future efforts should focus on bioinspired interface engineering (e.g., dynamic covalent bonds for toughness) and gradient/core–shell designs to harmonize mechanical-electroactive properties, alongside bio-derived dopants (natural polyelectrolytes, DNA) and metabolic tracking to reduce immunogenicity. Integrating multimodal smart-responsive systems (e.g., light-electro triggered drug release) and 4D-printed dynamic reconfiguration technologies could address tissue regeneration’s temporal demands. Concurrently, advancing scalable manufacturing (high-throughput spinning-polymerization) and clinical-grade validation are critical to resolving challenges in long-term electroactivity, degradation-regeneration synchronization, and vascularization, ultimately accelerating translation into precision applications like neural interfaces and dynamic cardiac patches. Furthermore, the application of PPy and its composites in stimuli-responsive systems may have considerable development, such as in the manufacture of electro-reactive microneedles. In addition, PPy can be incorporated into pH-sensitive materials to develop pH-responsive platforms. The device can release its cargo in response to pH changes in the external environment or within the cell. Research in this field may lead to the emergence of new ‘smart materials’ with better processability, improved mechanical properties, biocompatibility, and excellent tissue function.

## Figures and Tables

**Table 1:** Application of electrospun PPy composite nanofibers in bone engineering.

Fabrication Method	Composition of the Composite	Arrangement Patterns of Nanofiber Scaffolds	Cell Type	In Vitro Performance	Ref.
Electrospinning	PAN/PPy	Aligned and random	Mouse bone marrow mesenchymal stem cells	Alignment process increased the tensile strength of nanofibers 3.9-fold, while the tensile strain of nanofibers decreased by 78%	[29]
Electrospinning	PPy/chitosan/collagen/PEO	Randomly arranged	Fibroblast cells	Enhanced the conductivity up to 164.274 × 10^−3^ s/m	[45]
Electrospinning and plasma polymerization	PLA/HA/Pyrrole/Iodine matrices	Randomly arranged and porous	Mesenchymal stem cells	Presenting a variety of apparent pore sizes to allow for the passage of nutrients to bone cells. The increased cell proliferation and significantly improved cell viability	[46]
Electrospinning and electrodepositing	Nanohydroxyapatite/PBAT/PPy	Randomly arranged	MG-63 cell	More hydrophilic with improved cell differentiation	[47]
Electrospinning and electrodepositing	PBAT/PPy/nHAp	Randomly arranged	Osteoblasts	Long-term antibacterial property, bioactivity, and osteoinductivity	[49]
Electrospinning	PLLA/PPy	An oriented direction and bead-free morphology	Bone mesenchymal stromal cells	Accelerated the osteogenic differentiation of the seeded cells	[50]
Electrospinning and in situ polymerization	PPy/Fe_3_O_4_/PLGA	Randomly arranged	MC3T3-E1 pre-osteoblasts	Good biocompatibility, hydrophilicity, and thermal stability	[51]
Electrospinning and in situ polymerization	PCL/PPy	Randomly arranged	MC3T3-E1 cell	Exhibited enhanced cell adhesion, proliferation, and differentiation in electrical stimulation conditions	[54]
Electrospinning and in situ polymerization	PCL/PPy/PSS	Randomly arranged	Human mesenchymal stem cells	Enhance the differentiation toward osteogenic outcomes	[55]

**Table 2:** Application of electrospun PPy composite nanofibers in tissue engineering and regenerative medicine.

Fabrication Method	Composition of the Composite	Cell Type	In Vitro Performance	Ref.
Electrospinning	PPy coating (MWCNT/chitosan-g-polycarbamate)	S42 and PC12	Supports better viability, growth, and axon formation. Aligned fiber arrangement positively regulates cell response.	[30]
Electrospinning and electrodeposition	Chitosan/PPy coating poly-L-lactone/poly (ε-caprolactone)	PC12	Supports the viability, differentiation, and axon growth of cultured cells. Electrical stimulation improves genes (NF-L and Trk A).	[33]
Electrospinning	PPy-coated cellulose acetate butyrate	SH-SY5Y	The cell adhesion of the coating substrate is reduced. Aligned fibers and electrical stimulation positively regulate neurite outgrowth.	[34]
3D Printing, electrodeposition and electrospinning	PPy-coated silk fibroin	Rat Schwann cells	Supports good cell attachment, growth, and axon growth. Printing parameters seriously affect cell response.	[57]
Electrospinning	PPy-coated PLCL/silk fibroin	PC12	Showed better proliferation. Electrical stimulation improved cell response in terms of proliferation and differentiation phenotypes.	[61]
Electrospinning	PPy/PBAT	Neuro2A	Supports cell adhesion and axon growth.	[64]
Electrospinning	PPy-coated polyacrylonitrile	Rat neurons and glial cells	Forms cluster morphology and axon formation. Electrical stimulation induces cell proliferation and maturation.	[65]
Electrospinning	PPy-coated poly (ε-caprolactone)	Mouse Schwann cells	Supports cell growth.	[67]
Electrospinning	Polyornithine-coated polylactic acid/PPy	PC12	Supports good cell viability and growth. Electrical stimulation and fiber arrangement significantly improved the differentiated cell phenotype.	[74]

## Data Availability

No new data were created or analyzed in this study. Data sharing is not applicable to this article.
